# Molecular characterization and amplified ribosomal DNA restriction analysis of entomopathogenic bacteria associated with *Rhabditis* (*Oscheius*) spp.

**DOI:** 10.1007/s13205-015-0326-1

**Published:** 2016-01-14

**Authors:** Balakrishnan Geetha Sangeetha, Cheruvandasseri Arumughan Jayaprakas, Jinachandrannair Vijayakumari Siji, Moochattil Rajitha, Basheerkutty Shyni, Chellappan Mohandas

**Affiliations:** Division of Crop Protection, Central Tuber Crops Research Institute, Sreekariyam, Trivandrum, Kerala India

**Keywords:** *Bacillus*, *Enterobacter*, Entomopathogenic nematode, 16S rDNA

## Abstract

Bacterial strains associated with entomopathogenic nematodes (EPNs) *Rhabditis* (*Oscheius*) spp. were isolated from infected cadavers of *Galleria mellonella*. The obtained 18 isolates were subdivided into nine phylogenetically different genera based on comparative sequence analysis of their 16S rRNA genes. The isolates were affiliated to three different class namely γ-*proteobacteria* (*Enterobacter*, *Proteus*, *Providencia*, *Pseudomonas*, *Stenotrophomonas*), β-*proteobacteria* (*Alcaligenes*) and *Bacilli* (*Bacillus*, *Enterococcus*, *Lysinibacillus*). It was observed that Gram-positive strains (*Bacilli*) were more frequently associated with the EPN, whereas Gram-negative isolates were affiliated to six different genera with more genotypic diversity. Subsequently, all bacterial isolates used in this study were analyzed by amplified ribosomal DNA restriction analysis (ARDRA). Eight restriction endonucleases (*Cfo*I, *Hin*fI, *Rsa*I, *Dde*I, *Sau*3AI, *Alu*I, *Hae*III, and *Msp*I) were examined and a total of 15 different genotypes were obtained, forming two heterogenous main clusters after analysis by un-weighted pair-group method using arithmetic averages.

## Introduction

Entomopathogenic nematodes (EPNs) lead a symbiotic association with specific enterobacteria. *Xenorhabdus* and *Photorhabdus* are two genera of bacteria that are symbiotically associated with specific nematodes belonging to the families Steinernematidae and Heterorhabditidae, respectively (Poinar 1990). The nematodes invade the larvae of susceptible insects and penetrate to the hemocoel, where they release their symbiotic bacteria. The bacteria proliferates, kills the insect larvae, and promotes nematode reproduction by providing nutrients from the actions of degradative enzymes on the insect cadaver and by producing antibiotics that inhibit the growth of other microorganisms (Akhurst and Boemare 1990). A striking feature of *Xenorhabdus* and *Photorhabdus* is phase variation, which affects a large number of membrane-bound, intra and extracellular proteins and secondary metabolites (Akhurst 1996; Forst et al. 1997). Phase I variants are involved in the symbiotic relationship with EPN and are isolated from the non-feeding infective stage nematodes and from the body cavities of insects killed by these nematodes. No role in symbiosis has yet been determined for phase II, which is associated only with EPN under laboratory conditions. They represent one important part of the spectrum of biocontrol agents that are used to control insect pests of economically important crops. The importance of entomopathogenic bacteria (EPB) as source for the discovery of antibacterial and antifungal molecules has been studied in depth, as highlighted in various reviews (Paul et al. 1981; Webster et al. 2002; Bode 2009).

*Rhabditis* (*Oscheius*) spp. isolated from different agroclimatic zones of Kerala resembles EPN and was found to be effective for the control of areca nut spindle bug in the field (Mohandas et al. 2004). These were found to kill a number of important insect pests within 24–72 h in laboratory conditions. *Rhabditis* (*Oscheius*) sp. was also reported as biological control agent against rice yellow stem borer, *Scirpophaga incertulas* (Walker) (Padmakumari et al. 2007). Deepa et al. (2011) isolated twelve different strains of symbiotic bacteria from the surface sterilized infective juveniles (IJs) of *R.* (*Oscheius*) spp. and sequencing of 16S rDNA of these isolates revealed that they belong to seven different genera viz*. Acinetobacter*, *Bacillus*, *Comamonas*, *Stenotrophomonas*, *Achromobacter*, *Klebsiella* and *Brucellaceae*. Three diketopiperazines viz cyclo(l-Pro-l-Leu), cyclo(d-Pro-l-Leu) and cyclo-(d-Pro-l-Tyr) with antimicrobial activity were isolated from *Bacillus cereus* associated with a *Rhabditis* (*Oscheius*) sp. (Kumar et al. 2012a). Two stilbenes viz 3,4′,5-trihydroxystilbene and 3,5-dihydroxy-4-isopropylstilbene with antimicrobial activity were also isolated from the same bacterium associated with *Rhabditis* (*Oscheius*) sp. (Kumar et al. 2012b). Tryptophan containing diketopiperazines viz cyclo-(l-Trp-l-Pro), cyclo-(l-Trp-l-Tyr), cyclo-(l-Trp-l-Ile), cyclo-(l-Trp-l-Leu) and cyclo-(l-Trp-l-Phe) with antibacterial activity against human pathogenic bacteria were isolated from *Comamonas* *testosteroni* associated with *R.* (*Oscheius*) sp. (Kumar et al. 2014a).

A number of studies have been carried out on the phylogenetic status of bacteria associated with EPN using 16S rRNA gene sequencing. *Xenorhabdus* and *Photorhabdus* spp. appear to display a high monophyletic diversity. The 16S rRNA gene sequence analysis has placed the *Xenorhabdus/Photorhabdus* group within the gamma subdivision of the purple bacteria (*Proteobacteria*) (Rainey et al. 1995). In both genera, identification of new bacterial species is difficult because most strains are phenotypically very similar and fail to give positive results in many biochemical tests for identification (Boemare and Akhurst 1988). Amplified ribosomal DNA restriction analysis (ARDRA) proved to be useful for the classification of bacterial strains at different taxonomic levels and to analyze the genetic variability between bacterial isolates depending on selection of conserved or variable regions in the ribosomal genes (Swings 1996; Tiedje 1996). Brunel et al. (1997) conducted a study about the fast and rapid identification of *Xenorhabdus* and *Photorhabdus* spp. by restriction analysis of PCR amplification of 16S rRNA gene and phylogenetic dendrogram was also constructed by the neighbor joining method. Fischer-Le Saux et al. (1998) also reported the PCR ribotyping of *Xenorhabdus* and *Photorhabdus* isolated from the Caribbean region in relation to the geographic distribution of their nematode host based on 16S rRNA and cluster analysis. Clarridge (2004) has studied the impact of 16S rRNA gene sequencing for the phylogeny and taxonomy of bacteria. All isolated bacteria viz *Enterobacter aerogenes*, *E. hormaechei*, *E. cancerogenus*, *Stenotrophomonas maltophilia*, *Pseudomonas* *fulva/parafulva* were Gram-negative except *Staphylococcus succinus* (Lambert) (Gouge and Snyder 2006). In addition to *X. cabanillasii*, other bacterial strains were isolated from *Steinernema* *riobrave* and identified to species level by 16S rDNA sequences (Christen et al. 2008).

The EPB associated with *R*. (*Oscheius*) sp. represent an important source of bioactive molecules with antibacterial and anticancer activity (Kumar et al. 2014b). Moreover, earlier reports also suggest that the EPN associated bacterial isolates were found to kill a number of agriculturally important insect pests (Deepa et al. 2010). Here, we present the identification and diversity of bacterial isolates based on phenotypic characteristics, molecular phylogenetic analysis of 16S rRNA gene sequence and amplified ribosomal DNA restriction analysis (ARDRA).

## Materials and methods

### Isolation of bacteria from insect hemolymph

The most common insect host used for in vivo production of EPN is the last instar of the greater wax moth (*G. mellonella*), because of its high susceptibility to most nematodes, ease in rearing, wide availability and ability to produce high yields (Flanders et al. 1996; Ehlers 2001). Late instars of *G. mellonella* were placed on the surface of a filter paper in 35 mm Petri dishes. Individual nematodes were transferred onto the filter paper surface at a dose rate of 400 per Petri dish. All the dishes were sealed with parafilm, and then incubated at 25 °C for 24 h. Thereafter, the dead larvae were removed, rinsed in distilled water and surface sterilized with 70 % ethanol, and left for drying in a laminar air flow cabinet. Hemolymph obtained by dissecting dorsally between the fifth and sixth interstitial segments was collected with a sterile loop and streaked on nutrient agar (NA) plates (Woodring and Kaya 1988) and incubated at 28 °C for 48 h. The sources of the nematodes used in this study are listed in (Table 1).

**Table 1:** Geographic locations from where EPNs were collected

Bacterial isolate	Nematode	Location
SCI	*Rhabditis* (*Oscheius*) sp.	Mudavanmugal, Kerala
KL	*Rhabditis* (*Oscheius*) sp.	Korani, Kerala
KPG	*Rhabditis* (*Oscheius*) sp.	Kannur, Kerala
KY1	*Rhabditis* (*Oscheius*) sp.	Kaniyoor, Kerala
HY	*Rhabditis* (*Oscheius*) sp.	Bangalore, Karnataka
KAL	*Rhabditis* (*Oscheius*) sp.	Kallambalam, Kerala
352	*Rhabditis* (*Oscheius*) sp.	Trivandrum, Kerala
MA1	*Rhabditis* (*Oscheius*) sp.	Manampur, Kerala
D	*Rhabditis* (*Oscheius*) sp.	Thiruvananthapuram, Kerala
SBI	*Rhabditis* (*Oscheius*) sp.	Coimbatore, Tamilnadu
TN5	*Rhabditis* (*Oscheius*) sp.	Tirunelveli, Tamilnadu
TAH	*Rhabditis* (*Oscheius*) sp.	Alwarkurichi, Tamilnadu
MM3	*Rhabditis* (*Oscheius*) sp.	Maruthamalai, Tamilnadu
MM2	*Rhabditis* (*Oscheius*) sp.	Maruthamalai, Tamilnadu
F34	*Rhabditis* (*Oscheius*) sp.	Coimbatore, Tamilnadu
KK2	*Rhabditis* (*Oscheius*) sp.	Kanyakumari, Tamilnadu
BR1	*Rhabditis* (*Oscheius*) sp.	Bangalore, Karnataka
S	*Rhabditis* (*Oscheius*) sp.	Soharpur, Orissa

### Phenotypic characterization

Phenotypic characteristics of each colony were studied from the standard array of morphological characters such as form, margin, color, elevation of colony by using stereomicroscope (Carl Zeiss, Stemi 2000C, USA) under 40× magnification. Gram staining and spore staining specific for identification of unknown bacterial strains were performed using 24-h bacterial culture on nutrient broth. Various biochemical tests such as citrate, nitrate reduction test, ortho-nitrophenyl-β-galactoside (ONPG), indole, methyl red, Voges–Proskauer, urease, motility indole ornithine (MIO), oxidase and catalase test were conducted according to the standard procedure of Cappuccino and Sherman (1996) and were interpreted 24–48 h later. For carbohydrate fermentation tests, 1 % of each test sugar (glucose, glycerol, maltose, mannitol, sucrose, starch, fructose, cetrimide and lactose) in peptone water was used. All the chemicals for biochemical test were purchased from the Himedia Laboratories Limited, Mumbai, India. Biochemical properties of the isolates have been studied and compared with Garrity et al. 2005

### Amplification of 16S rDNA

Total genomic DNA of bacteria was extracted according to the protocol (Reinhardt et al. 2008). Bacterial 16S rDNA was amplified using bacterial universal primers: forward primer fD1 5′AGAGTTTGATCCTGGCTCAG3′ (corresponding to 8–27 of *E. coli*) and reverse primer RP2 5′CGGCTACCTTGTTACGACTT3′ (corresponding to 1492–1510 of *E. coli*) (Weisburg et al. 1991). PCR was performed in a final volume of 25 μl reaction containing 2.5 μl of 10× Taq buffer A (containing 15 mM MgCl_2_), 0.5 μl 10 mM dNTPs (2.5 mM each), 1.0 μl of each primer (20 ng), 2 μl of template DNA and 0.25 μl of (1U) Taq DNA polymerase (Bangalore GeNei, India). The amplification conditions were 92 °C for 2 min 10 s followed by 30 cycles of 1 min 10 s at 94 °C, 30 s at 49 °C and 2 min at 72 °C and followed by 10 min at 72 °C. The amplified products were resolved on 1 % agarose gel containing ethidium bromide 0.5 μg/ml. The DNA bands were visualized under UV transilluminator and documented with Gel Doc system (Alpha imager, Alpha Innotech, USA). DNA ladder of 500 bp (Bangalore GeNei, India) was used for determining the size of the amplicon.

The 16S rDNA gene sequences were generated by sequencing the PCR product on Applied Biosystems 3500 Genetic Analyzer (Big Dye Terminator v 3.1). The sequences obtained for the bacterial isolates were aligned with secondary-structure based Infernal aligner (https://rdp.cme.msu.edu/). The nucleotide sequences were compared with those in the NCBI databases using the Basic Local Alignment Search Tool (BLAST, http://www.ncbi.nlm.nih.gov/BLAST). From the aligned sequences phylogenetic tree was constructed using MEGA 6.06 software (Tamura et al. 2013).

The 16S rRNA gene sequences have been deposited in the NCBI Gen Bank with the following accession numbers: BR1 (JN712651), D (KJ578727), F34 (KJ600626), HY (KJ935725), KPG (JN982043), KK2 (JX470956), SBI (JX470955), KAL (JX470957), KY1 (JX470960), KL (KJ578730), MA1 (KJ600625), MM2 (KJ600624), MM3 (JN712652), S (JX470961), SCI (KJ600627), TN5 (KJ578729), TAH (KJ578730), 352 (KJ935726).

### Amplified ribosomal DNA restriction analysis (ARDRA)

For each isolate, 10 μl of PCR-amplified 16S rDNA was digested for 4 h at 37 °C with 5 U of restriction endonuclease *Cfo*I, *Hin*fI, *Rsa*I, *Dde*I, *Sau*3AI, *Alu*I, *Hae*III, and *Msp*I (Bangalore GeNei, India) according to the manufacturer’s instructions and separated by electrophoresis on 2 % agarose gels in 1× Tris–acetate–EDTA buffer for 2 h at 70 V. The gels were stained and photographed as described above. For each restriction enzyme’s band, a binary data matrix was constructed on the basis of the presence or absence of each band. The band patterns obtained with each enzyme were combined to obtain a single pattern for each isolate. Dendrogram was constructed by un-weighted pair-group method using arithmetic averages (UPGMA) with MEGA 6.06 software (Tamura et al. 2013).

## Results

### Phenotypic characterization

Morphological characters of colonies observed on NA plates were different for each bacterial strains and with entirely different biochemical characteristics from those exhibited by genera *Xenorhabdus* and *Photorhabdus* (Table 2). Pigmentation was not observed for the bacterial colonies. Colonies formed on NA plates were irregular, raised, convex or flat, white in color, with lobate, serrate or undulate margins. Correlation between strain characteristics and geographic locations where nematodes samples were collected was not observed in this study. Endospores were observed for the isolates BR1, HY, KPG, KY1, KAL, KK2, SBI and SCI on NA plates within 48 h of incubation at 30 °C. The spores were central or subterminal in position with one or two within a cell. All the strains gave a negative reaction for indole, ONPG, Voges–Proskauer test and positive reaction for methyl red and catalase test. It was also observed that biochemical characters exhibited by the isolates were different for each bacterial strains and most of the isolates gave variable responses for carbohydrate fermentation tests.

**Table 2:** Colony morphology and biochemical characteristics of the bacterial strains

Characteristic	SBI	BR1	KPG	KY1	KAL	KK2	HY	SCI	MM3
Shape	Rod	Rod	Rod	Rod	Rod	Rod	Rod	Rod	Rod
Form	Irregular	Irregular	Irregular	Irregular	Irregular	Irregular	Irregular	Irregular	Irregular
Margin	Lobate	Undulate	Lobate	Serrate	Serrate	Lobate	Undulate	Undulate	Serrate
Color	White	White	White	White	White	White	White	White	Creamish white
Elevation	Convex	Convex	Convex	Convex	Convex	Convex	Convex	Flat	Flat
Pigmentation	−	−	−	−	−	−	−	−	−
Gram stain	+	+	+	+	+	+	+	+	−
Motility	+	+	+	+	+	+	+	+	+
Spore formation	S	S	S	S	S	S	S	S	−
Citrate utilization	−	−	−	−	−	−	−	−	+
Nitrate reduction	+	−	−	+	−	−	−	−	−
ONPG	−	−	−	−	−	−	−	−	−
Indole	−	−	−	−	−	−	−	−	−
MR	+	+	+	+	+	+	+	+	+
VP	−	−	−	−	−	−	−	−	−
Urease	−	−	−	−	−	W+	W+	−	+
TSI (slant, butt, gas, H_2_S)	Alkaline/acid/−/−	Alkaline/acid/−/−	Alkaline/acid/−/−	Alkaline/acid/−/−	Acid/acid/−/−	Alkaline/acid/−/−	Alkaline/acid/−/−	Alkaline/acid/−/−	Alkaline/gas/−/−
MIO	+	+	+	+	+	+	+	+	+
Oxidase	+	+	−	+	−	−	−	+	+
Catalase	+	+	+	+	+	+	+	+	+
Glucose	+	+	+	+	+	+	+	+	+, Gas
Glycerol	−	−	−	−	−	W+	−	−	+, Gas
Maltose	+	+	+	+	−	+	+	+	+, Gas
Mannitol	−	−	−	W+	−	−	−	−	+, Gas
Sucrose	+	+	+	+	−	+	−	+	+
Starch	+	+	+	+	−	W+	−	+	W+
Fructose	+	+	+	+	+	+	W+	+	+, Gas
Cetrimide	−	−	−	−	−	_	−	−	−
Lactose	−	−	−	W+	−	_	−	−	+, Gas

Characteristic	TN5	352	S	MM2	MA1	TAH	KL	D	F34
Shape	Cocci	Rod	Rod	Rod	Rod	Cocci	Rod	Rod	Rod
Form	Circular	Irregular	Irregular	Irregular	Irregular	Circular	Circular	Irregular	Irregular
Margin	Entire	Undulate	Serrate	Serrate	Lobate	Entire	Entire	Lobate	Serrate
Color	Yellowish	White	White	Creamish white	White	Yellowish	White	White	White
Elevation	Raised	Convex	Convex	Flat	Convex	Raised	Convex	Convex	Convex
Pigmentation	−	−	−	−	−	−	−	−	−
Gram stain	+	+	−	−	−	+	−	−	−
Motility	+	+	+	+	+	+	+	+	+
Spore formation	−	−	+	−	−	−	−	−	–
Citrate utilization	+	**+**	−	**+**	+	+	+	−	+
Nitrate reduction	−	−	−	−	−	−	−	−	−
ONPG	−	−	−	−	−	−	−	−	−
Indole	−	−	−	−	−	−	−	−	−
MR	−	+	+	+	+	+	+	+	+
VP	−	−	−	−	−	−	−	−	−
Urease	+	W+	W+	−	+	−	+	+	−
TSI (slant, butt, gas, H_2_S)	Alkaline/acid/−/−	Alkaline/acid/−/−	Alkaline/acid/−/−	Alkaline/acid/−/−	Alkaline/−/−/H_2_S	Acid/acid/gas/−/−	Acid/acid/gas/−/−	Acid/alkaline/−/H_2_S	aicd/acid/gas/−/−
MIO	+	+	+	+	+	+	+	+	+
Oxidase	+	−	−	+	+	+	+	−	−
Catalase	+	+	+	+	+	+	+	+	+
Glucose	+	+	+	+	+, Gas	+, Gas	+, Gas	+	+
Glycerol	W+	−	−	W+	W+	W+	−	+	+
Maltose	W+	+	+	+	+, Gas	+, Gas	+, Gas	+	+
Mannitol	W+	−	−	W+	+, Gas	+, Gas	+, Gas	+	W+
Sucrose	W+	−	−	+	+, Gas	+, Gas	+, Gas	+	+
Starch	W+	+	−	W+	W+	W+	W+	−	+
Fructose	W+	+	W+	+	+	+, Gas	+, Gas	+	+
Cetrimide	W+	−	−	−	−	W+	−	−	−
Lactose	W+	−	−	W+	+	−	W+	−	W+

+ positive, − negative, *W+* weakly positive, *S* spore formation, *VP* Voges–Proskauer, *MR* methyl red, *MIO* motility indole ornithine, *Gas* gas production

The isolate MM2 and KL show similar phenotypic characteristics of *Alcaligenes faecalis* except for fructose, maltose fermentation test. *A. faecalis* does not produce acid but the isolates utilize fructose and maltose as sole source of carbon. Key positive characteristics for *Enterococcus faecalis* are non-motile, catalase negative and ferment glucose without gas production. The strains TAH and TN5 were motile and catalase positive, which indicates different strain characteristics. Biochemical characters of isolate 352 were similar to *Lysinibacillus sphaericus* but the strain showed positive reaction for fructose, maltose, starch and glucose fermentation test. *Stenotrophomonas maltophilia* were oxidase negative but MM3 isolate shows positive reaction for oxidase. Strain S shows negative reaction for citrate and positive for methyl red. But *Pseudomonas* strains always gave positive reaction for citrate and negative for methyl red. Isolates D, F34 and MA1 show similar phenotypic characteristics of *Proteus mirabilis*, *Providencia* sp. and *Enterobacter* sp., respectively. The colony morphological characters and the biochemical test results of all the isolates are shown in (Table 3).

**Table 3:** Identification of bacterial species associated EPN based on sequencing of 16S rDNA

Isolate	Identification	*E* value	Similarity (%)
SCI	*Bacillus thuringiensis* strain Bt407 16S ribosomal RNA, complete sequence (NR 102506)	0.0	98
SBI	*Bacillus cereus* strain ATCC 14579 16S ribosomal RNA gene, partial sequence (NR 114582)	0.0	99
KK2	*Bacillus cereus* strain ATCC 14579 16S ribosomal RNA (rrnA) gene, complete sequence (NR 074540)	0.0	99
HY	*Bacillus cereus* strain CCM 2010 16S ribosomal RNA gene, complete sequence (NR 115714)	0.0	99
KAL	*Bacillus cereus* strain NBRC 15305 16S ribosomal RNA gene, partial sequence (NR 112630)	0.0	98
KPG	*Bacillus cereus* strain JCM 2152 16S ribosomal RNA gene, partial sequence (NR 113266)	0.0	98
BR1	*Bacillus cereus* strain ATCC 14579 16S ribosomal RNA (rrnA) gene, complete sequence (NR 074540)	0.0	98
KY1	*Bacillus cer*eus strain IAM 12605 16S ribosomal RNA gene, partial sequence (NR 115526)	0.0	99
352	*Lysinibacillus sphaericus* C3-41 16S ribosomal RNA, complete sequence (NR 074883)	0.0	99
F34	*Providencia* stuartii strain MRSN 2154 16S ribosomal RNA, complete sequence (NR 102978)	0.0	98
D	*Proteus mirabilis* strain HI4320 16S ribosomal RNA, complete sequence (NR 074898)	0.0	97
MM2	*Alcaligenes faecalis* strain NBRC 13111 16S ribosomal RNA gene, partial sequence (NR 113606)	0.0	97
KL	*Alcaligenes faecalis* strain NBRC 13111 16S ribosomal RNA gene, partial sequence (NR 113606)	0.0	98
TN5	*Enterococcus faecalis* V583 strain V583 16S ribosomal RNA, complete sequence (NR 074637)	0.0	97
MM3	*Stenotrophomonas maltophilia* strain IAM 12423 16S ribosomal RNA gene, complete sequence (NR 041577)	0.0	99
S	*Pseudomonas monteilii* 16S ribosomal RNA, complete sequence (NR 121767)	0.0	99
MA1	*Enterobacter hormaechei* strain 0992 77 16S ribosomal RNA gene, partial sequence (NR 042154)	0.0	98
TAH	*Enterococcus faecalis* strain ATCC 19433 16S ribosomal RNA gene, partial sequence (NR 115765)	0.0	98

## 16S rDNA sequence analysis

**Fig. 1:**
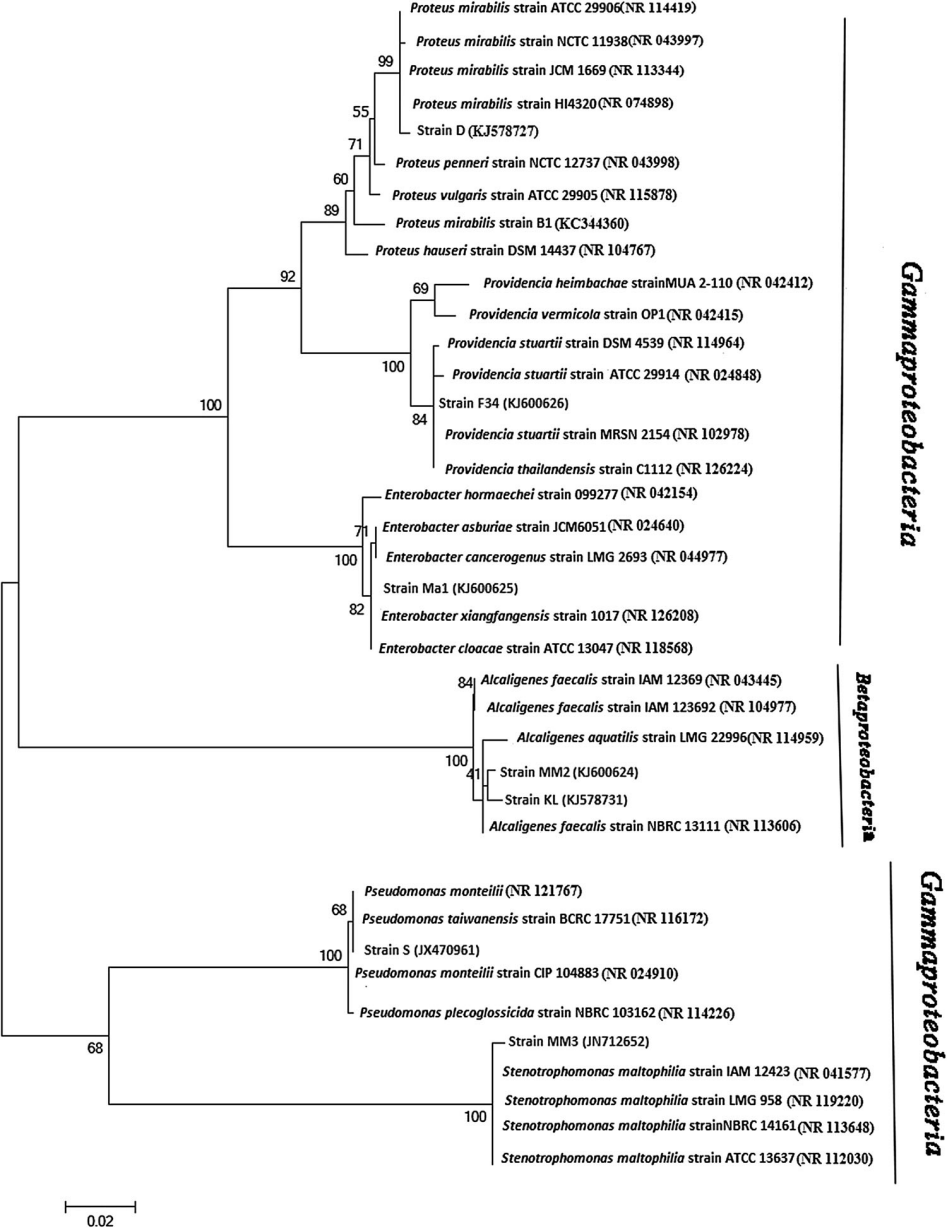
Phylogenetic tree inferred from 16S rDNA sequences analysis showing the relationships of *Rhabditis* (*Oscheius*) spp. associated EPB isolates with several members of the *Proteobacteria*

**Fig. 2:**
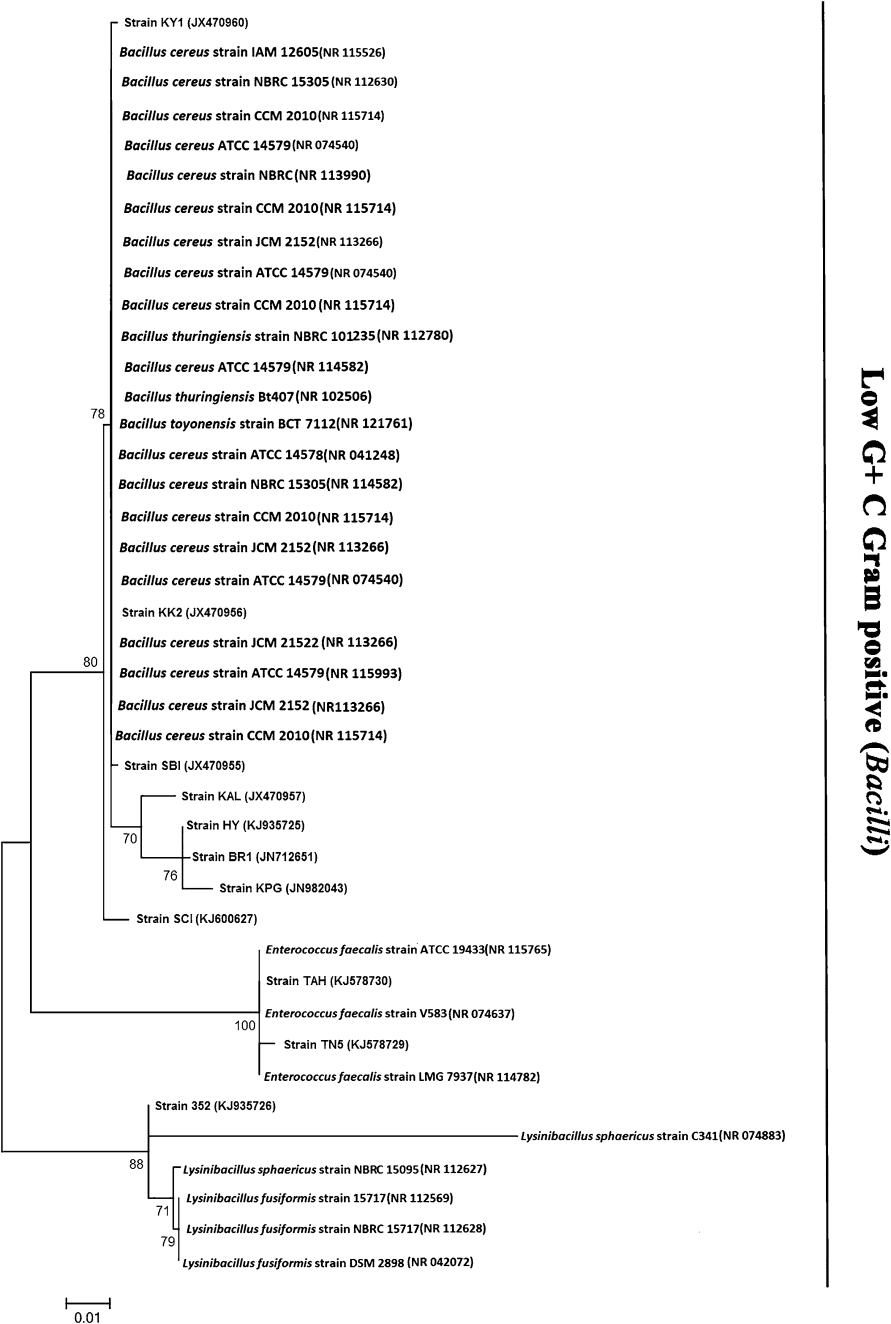
Phylogenetic tree inferred from 16S rDNA sequences analysis showing the relationships of *Rhabditis* (*Oscheius*) spp. associated EPB isolates with several members of the class *Bacilli*

Sequences with >98 % sequence similarity to their nearest phylogenetic neighbor were identified to the species level. The 18 different strains were affiliated to nine different genera by comparative analysis of 16S rDNA sequences. The isolates were identified as MM2 and KL-*A. faecalis*, TN5 and TAH-*E. faecalis*, D-*P. mirabilis*, 352-*L. sphaericus*, MM3-*S. maltophilia*, MA1-*Enterobacter* sp., F34-*Providencia* sp. and S- *Pseudomonas* sp. Sequence similarity analysis of the strains BR1, HY, KPG, KY1, KAL, KK2, SBI, and SCI shows that they were closely related to *B. cereus* with more than 98 % similarity. BLAST results were shown in Table 4. Phylogenetic relationship of Gram-negative and Gram-positive bacteria based on 16S rRNA gene sequences is shown in Figs. 1, 2.

**Table 4:** Comparison of morphological and biochemical characteristics of the bacterial strains with *Xenorhabdus* and *Photorhabdus* sp

Characteristic	SBI	BR1	KPG	KY1	KAL	KK2	HY	SCI	MM3
Shape	Rod	Rod	Rod	Rod	Rod	Rod	Rod	Rod	Rod
Form	Irregular	Irregular	Irregular	Irregular	Irregular	Irregular	Irregular	Irregular	Irregular
Pigmentation	−	−	−	−	−	−	−	−	−
Gram stain	+	+	+	+	+	+	+	+	−
Bioluminescence	−	−	−	−	−	−	−	−	−
Motility	+	+	+	+	+	+	+	+	+
Spore formation	S	S	S	S	S	S	S	S	−
Nitrate reduction	+	−	−	+	−	−	−	−	−
ONPG	−	−	−	−	−	−	−	−	−
Urease	−	−	−	−	−	W+	W+	−	+
MR	+	+	+	+	+	+	+	+	+
VP	−	−	−	−	−	−	−	−	−
Oxidase	+	+	−	+	−	−	−	+	+
Catalase	+	+	+	+	+	+	+	+	+
Glucose	+	+	+	+	+	+	+	+	+, Gas
Glycerol	−	−	−	−	−	W+	−	−	+, Gas
Maltose	+	+	+	+	−	+	+	+	+, Gas
Lactose	−	−	−	W+	−	_	−	−	+, Gas
Sucrose	+	+	+	+	−	+	−	+	+

Characteristic	TN5	352	S	MM2	MA1	TAH	KL	D	F34	*Xenorhabdus* sp.	*Photorhabdus* sp.
Shape	Rod	Rod	Rod	Rod	Rod	Rod	Rod	Rod	Rod	Rod	Rod
Form	Circular	Irregular	Irregular	Irregular	Irregular	Circular	circular	Irregular	Irregular	Irregular	Circular
Pigmentation	−	−	−	−	−	−	−	−	−	+	+
Gram stain	+	+	−	−	−	+	−	−	−	−	−
Bioluminescence	−	−	−	−	−	−	−	−	−	−	+
Motility	+	+	+	+	+	+	+	+	+	+	+
Spore formation	−	−	+	−	−	−	−	−	−	−	−
Nitrate reduction	−	−	−	−	−	−	−	−	−	−	−
ONPG	−	−	−	−	−	−	−	−	−	d	−
Urease	+	W+	W+	−	+	−	+	+	−	−	d
MR	−	+	+	+	+	+	+	+	+	−	−
VP	−	−	−	−	−	−	−	−	−	d	−
Oxidase	+	−	−	+	+	+	+	−	−	−	−
Catalase	+	+	+	+	+	+	+	+	+	−	+
Glucose	+	+	+	+	+, Gas	+, Gas	+, Gas	+	+	+	+
Glycerol	W+	−	−	W+	W+	W+	−	+	+	+, Gas	+
Maltose	W+	+	+	+	+, Gas	+, Gas	+, Gas	+	+	+, Gas	+
Lactose	W+	−	−	W+	+	−	W+	−	W+	_	−
Sucrose	W+	−	−	+	+, Gas	+, Gas	+, Gas	+	+	−	−

+ positive, − negative, *W+* weakly positive, *S* spore formation, *VP* Voges–Proskauer, *MR* methyl red, *Gas* gas production, *d* vary depending on species

### Amplified ribosomal DNA restriction analysis (ARDRA)

**Fig. 3:**
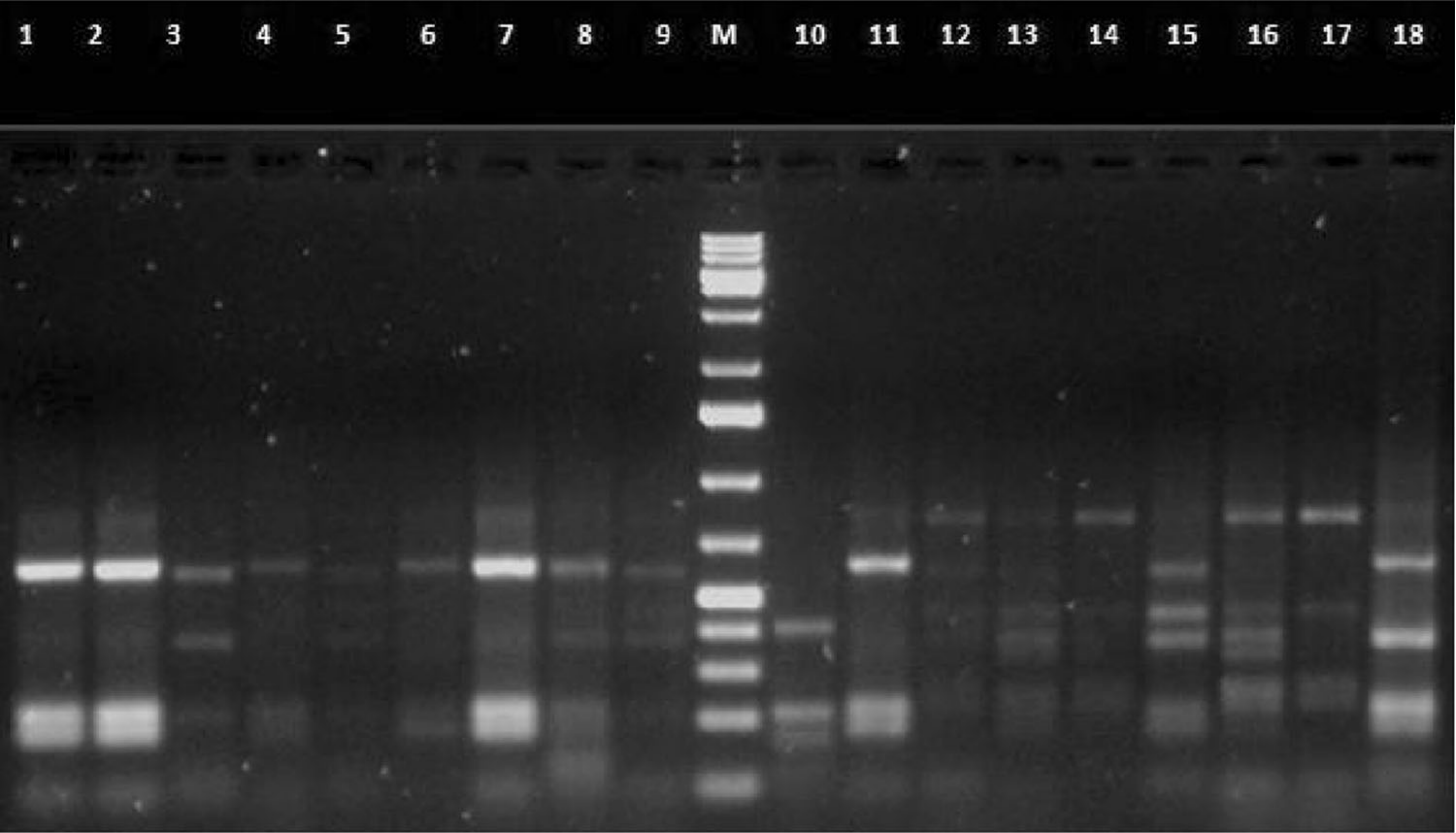
Restriction patterns of PCR-amplified 16S rRNA genes from bacterial strains digested with restriction endonuclease *Alu*I

**Fig. 4:**
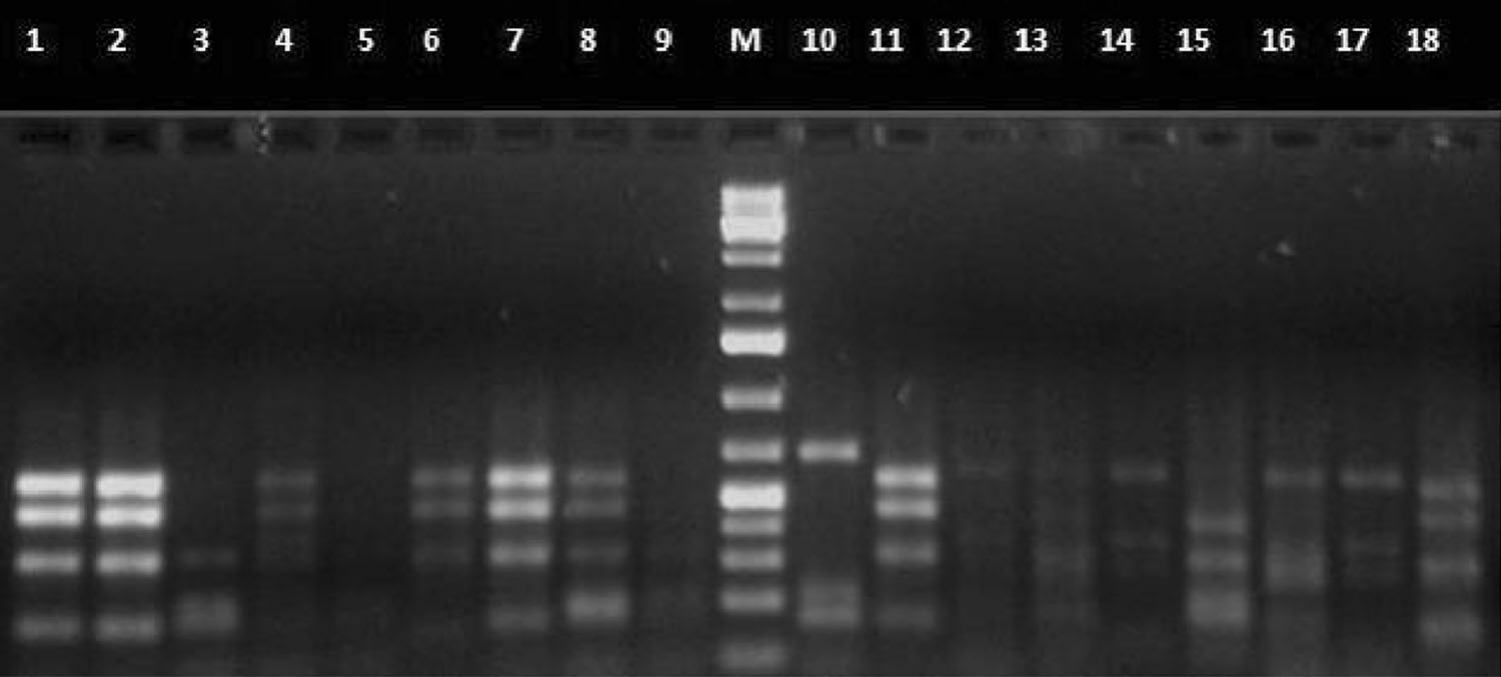
Restriction patterns of PCR-amplified 16S rRNA genes from bacterial strains digested with restriction endonuclease *Hae*III. *Lane* *1*–*8* *Bacillus cereus* KPG, BR1, KY1, SBI, KAL, SCI, KK2, HY, 9-*Enterococcus faecalis* TN5, M-1kbp plus ladder (Bangalore GeNei, India), 10-*Lysinibacillus sphaericus* 352, 11-*Pseudomonas* sp. S, 12-*Alcaligenes faecalis* MM2, 13-*Enterobacter* sp.MA1, 14-*Enterococcus faecalis* TAH, 15-*A. faecalis* KL, 16-*Proteus mirabilis* D, 17-*Providencia* sp. F34, 18-*Stenotrophomonas* sp.MM3

**Fig. 5:**
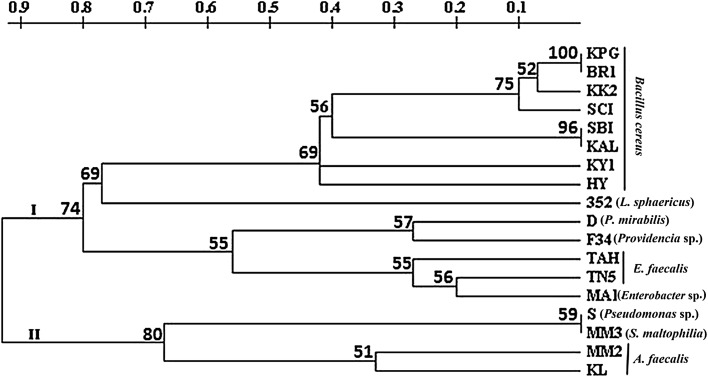
Dendrogram based on UPGMA cluster analysis obtained for the combined ARDRA restriction profiles

All the 18 isolates were further investigated by amplified ribosomal DNA restriction analysis (ARDRA). Polymorphic restriction patterns of PCR-amplified 16S rDNA were obtained with the *Alu*I and *Hae*III endonucleases. Results of the restriction analysis patterns are presented in Figs. 3, 4, respectively. A total of 15 genotypes were identified, forming two heterogenous main clusters based on dendrogram (Fig. 5) after analysis by un-weighted pair-group method using arithmetic averages (UPGMA).

## Discussion

The present study describes the molecular characterization of EPB isolated from *R.* (*Oscheius*) sp. and based on the 16S rDNA sequence analysis, the 18 isolates were identified as *Enterobacter sp.*, *P. mirabilis*, *Providencia sp.*, *Pseudomonas sp.*, *S. maltophilia* (class γ-*proteobacteria)*, *A*. *faecalis* (class β-*proteobacteria*) and *B. cereus*, *E. faecalis*, *L. sphaeriscus* (class *Bacilli*). The restriction analysis data of PCR-amplified 16S rDNA of the isolates revealed that they came under 15 different genotypes and were grouped into two heterogenous main clusters based on dendrogram.

In the current investigation, the EPB were isolated from EPNs *Rhabditis* (*Oscheius*) sp. which belongs to the family Rhabditidae. Zhang et al. (2008) and Chaston et al. (2011) opined that EPB are usually associated with EPNs of the family Steinernematidae, Heterorhabditidae and Rhabditidae. Gram-negative enterobacteria of the genera *Xenorhabdus* and *Photorhabdus* are the most studied mutualistic symbionts of the EPNs *Steinernema* and *Heterorhabditis* (Boemare et al. 1993; Forst and Clarke 2002; Chaston et al. 2011). The molecular and cellular interface between the host and the bacteria during the colonization process is the reason for unique nematode–bacterium association (Goodrich-Blair and Clarke 2007; Snyder et al.^2007^). The complete genome sequence data of *Xenorhabdus* and *Photorhabdus* sp. revealed a set of common genes encoding toxins, proteases, putative membrane transporters, transcriptional regulators and genes encoding lipopolysaccharide production, which help in stabilizing the nematode-bacterium mutualism (Chaston et al. 2011).

The EPB isolated in this study has been reported to have biocontrol activity (Deepa et al. 2010; Mohandas et al. 2007). Many nematode species such as (*Rhabditis* (*Oscheius*) sp., *Rhabditis blumi*, *Heterorhabditidoides chongmingensis*, *Heterorhabditidoides rugaoensis* of the family Rhabditidae have association with EPB and together act as potent biological control agent (Zhang et al. 2008, 2012; Park et al. 2012). The insect pathogenic bacteria viz *Providencia vermicola*, *A. faecalis* and *Flavobacterium* sp. isolated from *R. blumi* have pathogenic effect on major cruciferous vegetable pests such as *Plutella xylostella* L., *Artogeia rapae* L., *Mamestra brassicae* L. (Park et al. 2011, 2012). Out of the 18 strains isolated from the *Rhabditis* (*Oscheius*) sp. 11 strains were identified as *B. cereus*. Associations between endospore forming *Paenibacillus nematophilus*, and *H. megidis* have already been reported (Enright et al. 2003). A *Bacillus* sp. was also reported to have phoretic relationship with the EPN, *Heterorhabditis* sp. (Marti and Timper 1999). The insecticidal activity of *B. cereus* by producing insecticidal proteins named Vip during the vegetative phase which in turn causes pathogenicity to insects of order Lepidoptera and Diptera reported by To et al. (1975), Kaaya and Darji (1989), Warren et al. (1996). Kumar et al. (2014a) reported antimicrobial property of the metabolites isolated from *B. cereus* associated with *Rhabditis* (*Oscheius*) sp. *L. sphaericus* is the first report to have its association with IJs of EPN. Although *L. sphaeriscus* was reported as an entomopathogen and produces toxin like sphaericolysin leading to haemocoelic toxicity toward *Blattela germanica* and *Spodoptera litura* (Castagnola and Stock 2014). It was the first report of the bacterium to have its association with IJs of EPN. The 16S rRNA gene sequencing of the isolates TN5 and TAH shared 98 % similarity with *E. faecalis* (NR 074637 and NR 115765). Walsh and Webster (2003) also reported the occurrence of two bacterial species viz. *Acinetobacter* and *Enterococcus* from *Steinernema* in addition to *Xenorhabdus* symbionts.

The Gram-negative isolates associated with *Rhabditis* (*Oscheius*) sp. belong to six different genera of class γ-*proteobacteria* and β-*proteobacteria.* Similar studies also reported isolation of Gram-negative EPB of different genera such as *Provindencia* (*proteus*) *rettgeri* (Jackson et al. 1995); *Enterobacter gergoviae*, *Vibrio* spp., *Pseudomonas fluorescens* type C, *Serratia marcescens*, *Citrobacter freundii*, and *Serratia proteomaculans* (Gouge and Snyder 2006) from EPN. Association of γ-proteobacterium *Moraxella osloensis (*Moraxellaceae) with the nematode *Phasmarhabditis hermaphrodita* (Rhabditidae) was reported by Tan and Grewal (2001) but the bacterium was phylogenetically distinct from either *Xenorhabdus* or *Photorhabdus* (Enterobacteriaceae). Symbiotic association of *Serratia nematodiphila* (Enterobacteriaceae) with a newly described EPN, *Heterorhabditidoides chongmingensis* which belongs to family Rhabditidae was reported by Zhang et al. (2009). All these studies revealed the association of γ-*proteobacteria* with EPNs.

Brunel et al. (1997) opined that restriction analysis of PCR-amplified 16S rDNA is a very effective technique for distinguishing between the bacterial symbionts of EPNs. Amplified Ribosomal DNA Restriction Analysis (ARDRA) of 16S rDNA was used to distinguish the EPB strains associated with *Rhabditis* (*Oscheius*) sp. The isolates BR1, HY, KPG, KY1, KAL, KK2, SBI, and SCI were identified as *B. cereus* but six different restriction patterns were observed by this method. These results corroborate with the studies on *Xenorhabdus* and *Photorhabdus* conducted by Fischer-Le Saux et al. (1998). The isolates TN5 and TAH were identified as *E. faecalis,* but they also show variable restriction patterns. Detection of new genotypes and grouping of *Xenorhabdus* and *Photorhabdus* using PCR-restriction fragment length polymorphism (RFLP) and 16S rDNA were reported by Akhurst (1996) and Brunel et al. (1997). All the bacterial isolates used in this study were also grouped into two heterogenous main clusters where group I comprises all the *B. cereus*, *L. sphaericus*, *E. faecalis*, *P. mirabilis*, *Providencia* sp. and *Enterobacter* sp. strains, whereas group II comprises *Pseudomonas* sp., *S. maltophilia* and *A. faecalis.* The clustering of the isolates may be due to the absence of variation in 16S rRNA genes among different bacterial species (Fox et al. 1992). It can also be explained by their shared environment, including a common host, which promoted the exchange of genetic material between the bacterial isolates. Entomopathogens in general have a history of horizontal gene transfer events, shuffling toxin containing plasmids and pathogenicity islands between each other (Castagnola and Stock 2014). The present study also reveals a group of novel EPB which will be useful for production of bioactive metabolites effective against bacterial and fungal diseases of plants and animals.

## Conclusion

The detailed study on the metabolites produced by the novel bacteria associated with the EPN brings up a promising biocontrol agent which can manage pests and diseases in an ecofriendly manner.
